# Investigating assumptions of vulnerability: A case study of the exclusion of psychiatric inpatients as participants in genetic research in low- and middle-income contexts

**DOI:** 10.1111/dewb.12251

**Published:** 2020-01-14

**Authors:** Andrea C. Palk, Mary Bitta, Eunice Kamaara, Dan J. Stein, Ilina Singh

**Keywords:** bioethics, genetics, sub-Saharan Africa, psychiatry, research ethics, exploitation, informed consent

## Abstract

Psychiatric genetic research investigates the genetic basis of psychiatric disorders with the aim of more effectively understanding, treating, or, ultimately, preventing such disorders. Given the challenges of recruiting research participants into such studies, the potential for long-term benefits of such research, and seemingly minimal risk, a strong claim could be made that all non-acute psychiatric inpatients, including forensic and involuntary patients, should be included in such research, provided they have capacity to consent. There are tensions, however, regarding the ethics of recruiting psychiatric inpatients into such studies. In this paper our intention is to elucidate the source of these tensions from the perspective of research ethics committee interests and decision-making. We begin by defining inpatient status and outline some of the assumptions surrounding the structures of inpatient care. We then introduce contemporary conceptions of vulnerability, including Florencia Luna’s account of vulnerability which we use as a framework for our analysis. While psychiatric inpatients could be subject to consent-related vulnerabilities, we suggest that a particular kind of exploitation-related vulnerability comes to the fore in the context of our case study. Moreover, a subset of these ethical concerns takes on particular weight in the context of genetic research in low- and middle-income countries. At the same time, the automatic exclusion of inpatients from research elicits justice-related vulnerabilities.

## 1 Introduction

Psychiatric genetic research investigates the genetic basis of psychiatric disorders with the aim of more effectively understanding, treating, or, ultimately, preventing such disorders. To achieve statistical significance genome wide association studies (GWAS) require massive sample sizes. This necessitates extensive recruitment of individuals with the disorders in question. Given the challenges of recruiting research participants, the potential for long-term benefits of such research, and seemingly minimal risk,^1^ a strong claim could be made that all non-acute psychiatric inpatients, including forensic and involuntary patients, should have the opportunity to participate in such research, provided they have capacity to consent.

Several reasons may be given in support of this claim. First, it is likely that psychiatric inpatients systematically have the most severe forms of the disorder in question, and that these forms of the disorder may have a more specific biological underpinning.^2^ For example, one might argue that it is essential to include inpatients in biological research studies in order to increase the probability that genetic variants that predispose individuals to severe forms of the disorder can be properly identified and analysed. Second, and connected with the previous point, if more severe cases of disorder are excluded from such studies, then understanding of the pathophysiology of disorder may be undermined, resulting in delayed development of molecular targets that generate better treatments. Third, studies indicate that psychiatric inpatients themselves hold favourable attitudes towards participation in psychiatric research.^3^ Fourth, the principle of justice supports a right to not be automatically excluded, without adequate justification, from participation in such research.^4^

Despite these factors, there are tensions regarding the ethics of recruiting psychiatric inpatients into large-scale genetic studies. Such tensions are highlighted in the context of international research consortia, involving collaboration between multiple institutions and Research Ethics Committees (RECs), also known as Institutional Review Boards (IRBs),^5^ located in both high-income countries (HICs) and low and middle-income countries (LMICs). In particular, the requisite justifications for REC approval of protocols that seek to include psychiatric inpatients can be more stringent; therefore, inpatients are sometimes excluded as potential research participants for pragmatic reasons. Given that obtaining REC approval for the recruitment of outpatients is perceived to be more feasible than approval for inpatients, it is clear that there are underlying assumptions regarding differences between inpatients and outpatients that warrant consideration.

In this paper our intention is not to draw decisive conclusions on the ethics of including psychiatric inpatients as research participants; rather, we wish to elucidate the source of tensions regarding their inclusion in the context of psychiatric genetic studies in low-resourced settings. Moreover, our discussion is conducted from the perspective of REC interests and decision-making. We take these tensions to be primarily informed by underlying assumptions of vulnerability; but also, possibly, by normative beliefs regarding inpatient care structures and uncertainty regarding ethics and research oversight in LMICs.

We start by defining inpatient status and outline some of the assumptions surrounding the structures of inpatient care. We then introduce contemporary conceptions of vulnerability. In the next section, we draw on Florencia Luna’s account of vulnerability as a framework for our analysis. While psychiatric inpatients could be subject to consent-related vulnerabilities, we suggest that a particular kind of exploitation-related vulnerability comes to the fore in the context of our case study. Moreover, a subset of these ethical concerns takes on particular weight in the context of genetic research in LMICs. At the same time, there are justice-related vulnerabilities associated with the automatic exclusion of inpatients from research.

## 2 Defining Inpatient Status

The term ‘psychiatric inpatient’ encompasses varying levels of need and care. The National Health Service (NHS) in the United Kingdom uses the term with reference to persons who require ‘hospital beds’ and access to 24-hour care or monitoring by various health care professionals.^6^ This includes voluntary admissions as well as persons who have been detained or admitted involuntarily. Levels of care include intensive, mandatory care in secure wards for patients deemed to be at risk to themselves or others, and care for those in an acute phase of psychiatric illness and longer-term treatment. The latter would encompass residential care, which may be in a psychiatric hospital or institutional environment, depending on the context. Further distinctions may also be made between low, medium and high secure inpatient care which is provided to forensic inpatients.

Inpatients may be contrasted with outpatients, referring to persons who do not require ‘bed-use’ or overnight care and who may or may not have received care as inpatients in the past. As is the case with inpatients, outpatients do not represent a homogeneous group but may be distinguished on the basis of varying levels of care-needs. Such persons may reside relatively independently in accommodation that has been provided for them, they may receive home-based support services, or they may attend a clinic or hospital for daily or weekly treatment. From the point of view of clinical practice, one assumes that a distinction between inpatients and outpatients generally holds: the symptoms of inpatients are more severe than those of outpatients. However, given the lack of homogeneity in these ‘groups’ and other contextual factors such as adherence to treatment regimes, this cannot be taken as self-evident. We will return to this point further below.

It is clear from the above that the term ‘inpatient’ may be conceptualized in various ways. It includes hospitalization, characterized by varying lengths of stay, and longer-term institutionalization in a dedicated psychiatric facility. Moreover, the nature of hospitalization or institutionalization will depend on context. However, the literature has, and continues to, largely describe psychiatric inpatients in the context of ‘total institutions’.^7^ In a recent review investigating how the term ‘psychiatric institutionalization’ is, and has been, conceptualized over time, Chow & Priebe identify four dominant interpretations.^8^ First, in keeping with Goffman’s influential work on the nature of institutions,^9^ the term was traditionally associated with the physical edifice and space in which inpatients are housed and contained. Second, contemporary conceptions associate it with the legislation that serves to contain and protect inpatients, thus restricting their freedom. Third, psychiatric institutionalization is conceptualized in terms of the authority and various forms of responsibilities that clinicians and health professionals involved in care-giving have, in meeting the needs of inpatients. This would include providing treatment, meeting basic daily needs as well as affording protection and stability. Finally, the term is associated with the way in which patients become habituated by longer-term institutional care, frequently developing particular conforming behaviours.^10^

Chow and Priebe’s review suggests that normative assumptions regarding institutionalization, along with evidence showing improved outcomes of patients who have been discharged from institutional to community-based care,^11^ have played a major role in the deinstitutionalization movement.^12^ Such assumptions, which largely play to a notion of inpatients as removed from society; trapped, powerless and manipulated, may have a lingering impact on perceptions regarding the ethical permissibility of recruiting inpatients into research studies. While the history of psychiatric institutionalization involves a multitude of serious ethical transgressions, the moral status of the contemporary psychiatric inpatient should not be automatically rendered via an historical lens. At the very least, contemporary inpatient facilities, and inpatients themselves, deserve an opportunity to update the historical picture. The old asylum had many negative features: isolation, dependency and loss of freedom in a paternalistic setting; but it also held a potential for safety, protection, care and structure that was recognized by Tuke and Pinel, the Quaker reformers.^13^ More recently, Sisti *et al* have drawn attention to the negative implications of the deinstitutionalization movement for persons with severe or treatment resistant mental disorders, for whom community-based care may be unsuitable.^14^ Such individuals frequently face homelessness or prison on account of a lack of safe, stable and humane care.^15^

## 3 Contemporary Conceptions of ‘Vulnerability’

In research contexts, the concept of vulnerability has traditionally been used to refer to persons or groups of persons who, due to inherent qualities or contextual factors, are considered to be more susceptible to being exploited, harmed or wronged in some way, and who thus warrant special protection as research participants.^16^ Psychiatric inpatients, in particular, have historically been afforded heightened protection in most research ethics guidelines on the basis that they constitute a so-called ‘vulnerable population’.^17^ In the case of psychiatric inpatients, particularly those with psychotic disorders, ascriptions of vulnerability have largely been informed by the concern that neuropsychological impairments and symptoms associated with their disorders impact their decisional capacity, posing challenges to securing their informed consent.^18^ Their dependent status as institutionalized or hospitalized persons is also regarded as a source of vulnerability that may impact their ability to protect their own interests.

Given the exploitation and harm of various groups in research contexts in the twenty first century, there are compelling reasons for having ascribed the status of vulnerability at group or subpopulation level. There has, however, been a concerted move towards more nuanced approaches to vulnerability that frame it in terms of particular contextual characteristics, sources or layers that contribute towards increased risk of exploitation or harm rather than conceiving it primarily in terms of qualities intrinsic to persons or groups, or solely as mediated through decisional capacity.^19^ The recent revision of the Council of International Organizations of Medical Sciences (CIOMS) and the World Health Organization (WHO) *International Ethical guidelines for health-related research involving humans,* evidences this move.^20^ Factors that have informed this shift are, among others, the recognition that there is diversity and intersectionality within particular groups or populations, previously regarded as uniformly vulnerable which has implications for the level of risk posed by research participation. There has also been a concomitant realization that vulnerability is informed by dynamic contextual factors and therefore cannot be determined *a priori;* and cognizance of the stigmatizing effects of stereotyping group ascriptions. This move away from a categorical approach to vulnerability is also informed by recognition of the importance of challenging paternalistic assumptions regarding impairment, disability, and competence due, in part, to the deleterious impact that such assumptions have on the lives of the persons in question. Much of the progress achieved in this area may be attributed to the increased participation in such discussions, of persons living with disabilities or impairments.^21^

Despite various conceptual disagreements^22^, the weight of what references to vulnerability aim to capture is undeniable.^23^ Genuine cognizance of vulnerability is a ‘moral safeguard’ that medical research cannot do without.^24^ A tension thus remains between the nuanced and contextual approach to vulnerability and inclusivity and the fundamental conviction that particular research participants do, nevertheless, require heightened protection from potential harm. In other words, while the former serves as a guiding ideal that has gained major traction, the latter remains the primary obligation of RECs in their appraisals of research protocols. This entails the inevitability of some form of reference to groups of persons in contexts that may be assumed to be indicative of vulnerability. In this regard, the CIOMS guideline 15 flags psychiatric inpatients as requiring special protection when considered as research participants.^25^

## 4 Luna’s Layered Approach to Vulnerability

Given various challenges involved in defining and applying the concept of vulnerability in different research contexts,^26^ it is clear why the use of taxonomies or typologies of vulnerability may be appealing for RECs. However, relying solely on the use of a taxonomy of vulnerability may foster a rigid or ‘check the box’ approach, whereby additional sources of vulnerability, as well as the dynamic, contextual interplay between various sources, are neglected.^27^ In fact, it has been argued that vulnerability is inherently and “irreducibly contextual”, thus necessitating its assessment by RECs in research protocols to be approached on a case by case basis.^28^ Despite the various conceptual disagreements discussed above, vulnerability must nevertheless do practical, protective work in research contexts. To this end, Luna provides one of the most comprehensive frameworks for approaching assessments of vulnerability, by which its structure is conceived of in metaphors of layers.^29^ Her framework is able to encompass various definitions and taxonomies – the ‘content’ or characteristics of vulnerability – as potential layers of vulnerability that “may be acquired, as well as removed, one by one...[or that] may overlap...[and which ‘function’ in] a relational and dynamic” manner, rather than as a quality that is static and intrinsic to persons or groups of persons.^30^

The first step in assessing a research protocol would therefore be to identify all the layers of vulnerability by using various definitions and taxonomies. It would then be necessary to rank the identified layers, with those vulnerabilities that have the most harmful potential consequences and are most likely to be actualized, assuming urgent status. Regarding the latter point, it is important to highlight the fact that vulnerability is similar to the notion of risk or probability in that it is a property or state that may or may or may not be actualized. Whether or not a vulnerability leads to an actual harm or wrong will depend upon the presence of a ‘stimulus condition’ or trigger.^31^ For example, a person with a psychiatric disorder may be vulnerable to harm in contexts where mental disorders are stigmatized, however, in a context where there is an inclusive and accepting view of mental disorders, this particular vulnerability will remain latent. Thus, the next step would be identifying which layers are most likely to be triggered in a research context by identifying the stimulus conditions for each layer.

The final step of the process would be to identify what Luna refers to as ‘cascade layers’.^32^ A cascade layer is a vulnerability which may create further layers of vulnerability or worsen existing ones. While cascade layers may be triggered by dysfunctional or ‘pathogenic’ social responses that exacerbate an existing vulnerability, they are not necessarily limited to the latter.^33^ For example, a student with an undiagnosed psychiatric disorder may find that her symptoms impact her ability to focus on her studies with the outcome that she fails and must leave university. This could then further exacerbate her symptoms, which could then impact her personal relationships leading to isolation and so on. While the original layer of vulnerability, her lack of diagnosis, could be attributed to a dysfunctional or ‘pathogenic’ context in some way, for example a shame-response due to the stigmatization of mental illness and thus avoiding seeking help, there could be non-pathogenic reasons for it. Cascade layers are generally associated with compound harmful effects^34^ and would thus assume priority in the process of ranking, particularly those cascade layers with clear and present stimulus conditions. Assessing a research context in this way requires reflexivity, in order to ensure that the emphasis is on minimizing and addressing layers of vulnerability rather than seeking to avoid them entirely through overprotection or exclusion which may inadvertently create additional layers of vulnerability, given any benefits that participation may hold.

## 5 Identifying Layers of Vulnerability

### 5.1 Consent-related layers of vulnerability

Using Luna’s approach, we turn first to identifying the layers of vulnerability presented by genetic research contexts. Given the prevalent association of research vulnerability with a compromised ability to protect one’s own interests, we start with consent-related layers of vulnerability.

As mentioned above, the REC obligation to ensure that psychiatric inpatients receive special protection in research contexts is largely informed by the assumption that their decisional capacity is impaired in some way.^35^ Studies have consistently evidenced higher levels of impairment in the decisional capacity of persons with particular psychiatric disorders, such as schizophrenia, in comparison with control participants.^36^ However, there is extensive inter-individual variation; many persons with schizophrenia, for example, obtain scores for decisional capacity that are comparable with control participants.^37^ Furthermore, various studies have shown that certain educational interventions are able to improve the decisional capacity of persons with psychotic disorders,^38^ in some cases, to levels comparable to control participants.^39^ A recent study conducted in South Africa found that the San Diego Brief Assessment of Capacity to Consent (UBACC), in conjunction with an iterative learning approach, led to improved understanding of genetic research of both persons with schizophrenia and control participants.^40^ Simply put, the assumption that the decisional capacity of persons with psychotic disorders renders them less able, as a group, to consent to participate in research is not based on conclusive evidence and will, it seems, depend on the potential research participant in question.

A study which permits the participation of psychiatric outpatients, while excluding inpatients indicates an assumption of some ethically relevant difference between the two groups. If this difference lies in their decisional capacity, we must then ask if there is evidence that inpatients responding to antipsychotic treatment have lower decisional capacity than outpatients responding to treatment. As noted in the previous paragraph, there is extensive inter-individual variation in the cognitive competencies, and thus the decisional capacities, of patients with psychosis. However, due to the disease process there is also intra-individual variation as the cognitive abilities of such patients may fluctuate throughout their lifetime, and even during the course of admission, depending on the nature of their disorders and the success of their treatment.^41^ Moreover, as cognitive competencies in the population are situated on a spectrum, it may be possible that at certain points, some outpatients have lower competencies than inpatients. This could be attributable not only to the above factors but also to the fact that competency is informed by context to a large extent. For example, inpatients may be in a more stable condition as they are adhering to their medications as well as receiving specialized support and shelter, among other factors.

In terms of REC exclusion of psychiatric inpatients from research protocols on the basis of decisional capacity concerns, this could be interpreted as indicating contradictions in REC decision-making processes. If a particular instrument has been approved by an REC as a non-biased measure of capacity to consent, this would imply that the REC deems the instrument to be a reliable means of determining suitability for research participation. We can then question why such a test may not also be used for inpatients to determine their capacity to consent to participation in a study. In cases where inpatients are omitted in the recruitment of a potential research sample, this means that they are not provided the opportunity to undergo an REC-approved test for capacity to consent to research participation. The omission of this group could be viewed as a straightforward vulnerability bias;^42^ or, it could be viewed as a contradiction of the REC process.

### 5.2 Exploitation-related layers of vulnerability

Despite the points discussed above, it is unlikely that RECs are guilty of flawed reasoning, or of failing to acknowledge the reliability of measures of decisional capacity in some way. What is more likely is that concerns regarding consent are not the primary motivating factor in cases where psychiatric inpatients are excluded from genetic research. The underlying assumptions informing such a decision are more likely to be informed by conceptions of vulnerability as susceptibility to exploitation, where the latter is interpreted in terms of ‘taking unfair advantage’ of a party. Exploitation is generally associated with research that has an unfair distribution of benefits and burdens.^43^ The concept has been described as having paradoxical qualities, however, as it is accepted that a situation or exchange may be exploitative even if both parties seemingly benefit in some way from it – i.e. where there is no obvious harm or burden incurred – and the consent of the exploited party has been secured.^44^ The example of persons working for wages that are extremely low by global standards, in contexts where these wages serve as a means of escaping extreme poverty and are therefore viewed in a positive light by the persons in question, is frequently given to illustrate this point.^45^

The most obvious concern regarding exploitation lies in one of the justifications for including psychiatric inpatients in genetic research. The difficulties in recruiting large numbers of cases of the disorders in question provide a strong motivation for including inpatients. However, it is because inpatients represent a so-called convenient or at-hand population that could be of great assistance in genetic research, that exploitation concerns come to the fore. Potential inpatient research participants may be vulnerable to “juridic vulnerabilities”^46^ if the institution in which they reside has a vested interest in the research, and thus, in the participation of inpatients. In addition, inpatients, and involuntary inpatients in particular, would be at risk of “deferential vulnerabilities”.^47^ Deferential vulnerability is highlighted in power disparities between caregivers and inpatients. The concern here is for potential coercion or abuse of trust, even if this is unintended. Trust may have been established between caregivers and inpatients; thus, if caregivers approach patients to inform them about a study they might be more likely to consent on this basis. Any inpatient might feel that if they refuse to participate this will affect their treatment outcomes, but involuntary and forensic patients may additionally assume that cooperation and participation in research would reflect favourably on their progress. Involving inpatients as research participants also heightens the risk of therapeutic misconceptions which elicit both consent- and exploitation-related layers of vulnerability. Among the factors predisposing potential study participants to the risk of therapeutic misconception are studies where the design involves activities used in clinical care (e.g. drawing blood or saliva) and where a participant has limited scope of available therapeutic care as is the case with psychiatric conditions.

Power disparities indicative of potential exploitation may be present not only between researchers and participants, but also at a macro or structural level.^48^ In fact, multinational research has been highlighted as a context indicative of potential exploitation of vulnerable populations, groups or persons.^49^ The potential for exploitation in such cases is heightened if funding and oversight originate from HICs and research takes places in LICs.^50^ It is likely that RECs in HICs are sensitive to this, and that reticence regarding the inclusion of inpatients in research could be informed, in part, by a desire to avoid appearing exploitative. It may also be that a well-intentioned avoidance of psychiatric inpatient recruitment is based on incorrect understanding of local contexts. A qualitative study that included members and chairs of 46 RECs in the United States assessed views and beliefs regarding challenges to research integrity in the developing world.^51^ The findings indicate that REC decision-making is challenged by a lack of locally relevant contextual knowledge and uncertainties regarding the quality of ethics and research oversight and infrastructure in developing countries, among other factors.^52^ It is likely that these factors and perceptions would play a role in decisions regarding whether or not to include psychiatric inpatients in research studies.

### 5.3 Special considerations in low- and middle-income contexts

In addition to the layers of vulnerability discussed above, the recruitment of inpatients into genetic studies in LMICs introduces further layers of vulnerability. A consent-related layer of vulnerability must be considered in the case of inpatients who subscribe to more communitarian or collective worldviews. Communitarian or collective worldviews, which are prevalent in many LMICs across Asia and Africa, may be distinguished from various strands of Western liberal individualism. While the latter emphasises the individual as self-determined, collective or communitarian worldviews hold in common the view that the self is constituted through relationality with others and in the context of a community. In African contexts, for example, this frequently translates into shared decision-making through consulting and deliberating with family, community members or elders. In such contexts, informed consent in research is a relational rather than an individual process.^53^ While the beliefs and values described as African or communitarian are characterized by diversity, in a manner akin to those regarded as Western or liberal individualist, there are nevertheless certain underlying commonalities that are the basis of the coherence of categorization. A common thread in African moral thought is the conception that personhood is, *and should be,* relationally acquired and maintained.^54^ By virtue of being in an institution, at a spatial distance from their families and communities, psychiatric inpatients who subscribe to such a worldview may not be able to consult with others as easily as they would if they were based in a community. Participation in genetic research, in which the informed consent process is immediately followed by the provision of a sample, may require restructuring in the case of inpatients, so as to enable the option of consultation with visiting family or community members.

The assumption that there is an ethically relevant difference between inpatients and outpatients in terms of suitability for recruitment is also challenged by various contextual factors in low-resourced settings. Outpatients may face socioeconomic challenges that impact their adherence to treatment regimes. They may, in fact, be subject to layers of vulnerability not shared by inpatients who are stabilized and responding to treatment. A person with limited financial resources who must utilise public transport which may be unreliable or unsafe, and then wait in long queues at a day clinic for her medication, faces challenges in maintaining her treatment which would not be present in the case of an inpatient who is receiving on-site care. South Africa has witnessed a particularly devastating example of this with the Life Esidimeni tragedy.^55^ In 2015 the South African government ‘deinstitutionalised’ over 1300 psychiatric patients by ending their contract with the Life Esidimeni group. These patients were then placed in the care of their families or various nongovernment organisations (NGOs) who were not equipped to provide the requisite specialist care. As a result of neglect and starvation, 143 of these patients subsequently died. Layers of vulnerability were triggered by the fact that these patients’ interests were subsumed by larger structural and political agendas. This example illustrates the way that assumptions about vulnerability that may hold in HICs cannot be generalized to resource-strained contexts: layers of vulnerability cannot be identified or addressed without thorough consideration of contextual factors.

In low-resourced contexts ‘allocational vulnerabilities’ that elicit exploitation concerns may be intensified. In research studies, allocational vulnerabilities come to the fore if participants are “lacking in subjectively important social goods”.^56^ In such contexts, any compensation such as a payment for participation, or perceived advantages such as being able to skip long queues at a health care facility may be regarded by some as an undue inducement to participate.^57^ The risk of undue inducement to participate in research is present for both inpatients and outpatients, however it may be greater for outpatients who must attend a clinic for treatment. This is another example of how the assumption that inpatients are more vulnerable, across the board, than inpatients, may be challenged by contextual factors.

Psychiatric inpatients could, however, be more susceptible to social or pathogenic layers of vulnerability in contexts where mental illness is stigmatized, as is the case throughout Africa.^58^ While participation in genetic research that involves providing a saliva or blood sample may not elicit any immediate or obvious risk or harm, social vulnerabilities associated with how research information is interpreted, could be triggered. Biogenetic models of mental disorder are frequently interpreted in reductionistic ways leading to essentialist or determinist thinking.^59^ Given the complexity of causal attributions of stigma, it is unclear whether or not such interpretations lead to a definitive increase or decrease in stigma; however, a systematic review of 33 studies conducted in HICs and LMICs found that, for the most part, biogenetic explanations of mental disorder did not increase acceptance.^60^ The risk of pathogenic layers of vulnerability associated with stigmatizing interpretations of genetic research is not specific to inpatients but applies to all research participants with psychiatric disorders. However, while it is unclear how inpatients could be more at risk of some form of stigma-related harm due to their participation in genetic research, it could be that insofar as their disorders *are* more severe than outpatients, this risk could be higher.

### 5.4 Injustice-related layers of vulnerability

As mentioned in the introduction, a particular layer of vulnerability facing psychiatric inpatients arises from the fact that researchers are aware that obtaining REC approval of protocols that seek to include such groups can be more stringent and time-consuming. Inpatients are therefore sometimes excluded as potential research participants for pragmatic reasons. In this way there is a risk that the interests of inpatients, in terms of their right to participate in research, may be subsumed by the pragmatic goals and interests of institutions. While inpatients do require special protection when included as research participants, special protection should not be taken to imply exclusion. Active exclusion of persons with psychiatric disorders from medical research in general “represents an under-recognized and worrisome cause of health inequity” and may therefore be unjust and thus unethical.^61^

An even stronger claim can be made in the case of genetic research, which is ultimately aimed at increasing understanding of the genetic basis of psychiatric disorders so as to better treat, or, ultimately, prevent such disorders. Given the sometimes devasting effects that these disorders have on the lives of persons, participating in such research entails being able to make an active contribution to this goal.^62^ A study of the views of inpatients with schizophrenia supports this claim as it found that they regarded “helping others and helping science” as valid reasons for participation, along with “the feeling of hope” afforded by participation in such research.^63^ Decisions about the inclusion of inpatients in research studies should take into account the stated desires of inpatients themselves, and this must be balanced against perceived goods of exclusion from research. For example, insofar as there is a low probability of genetic research leading to improved treatments for persons with psychiatric disorders, participation in such research could be burdensome.^64^ However, the choice to participate should not be denied to persons in the name of protection or as a way of avoiding the logistical challenges associated with their inclusion.

### 5.5 Ranking layers and identifying stimulus conditions and cascade layers

We now turn our attention to identifying the stimulus conditions of the layers of vulnerability discussed in the previous section. Looking first at the issue of consent, it is clear that any consent-related vulnerabilities would be triggered if consent is not genuine, possibly due to therapeutic misconceptions, or if it is given without being able to consult with others if this is desired. Consent-related vulnerabilities could also be triggered by faulty or unethical research protocols or practices. However, these layers of vulnerability would remain latent if a reliable test for decisional capacity is used to ensure that those who do give consent are able to do so and fully understand the nature of their involvement; if ethical oversight is robust and researchers are sufficiently trained; and if prospective participants are given the option to consult with family members.

Looking at the various layers of vulnerability associated with increased likelihood of exploitation, there are a number of possible stimulus conditions. Deferential vulnerability would be triggered if caregivers approach patients to participate in research and fail to convince them that non-participation will have no negative consequences. Juridic vulnerabilities could be triggered if caregivers are also involved with a research project. This risk is particularly high if pressure to meet recruitment quotas impacts the recruitment process in ethically problematic ways. Allocational vulnerability would be triggered if payments to inpatients are excessively high, or if potential participants are made to feel that they will be treated favourably in some way. While the latter kind of allocational vulnerability may not be explicitly stated or even overtly experienced, there does need to be reflexive awareness among researchers such that any wrong assumption of preferential treatment on the part of inpatients can be corrected. Juridic and allocational vulnerabilities could also be triggered in contexts in which adequate ethical oversight is lacking, while deferential vulnerabilities could persist even if research practices are ethical and sensitive to such possibilities. In our view, vulnerability related to exploitation should assume priority because it can take more subtle forms, and is therefore most likely to be actualized despite ethical oversight.

In Luna’s framing of vulnerabilities, it is undeniable that having a psychiatric disorder, particularly one that is sufficiently serious to warrant inpatient care, is a potential cascade layer of vulnerability. Certain layers of vulnerability associated with being a psychiatric inpatient are ubiquitously present, such as the potential for fluctuation in decisional capacity. However, we would argue that the likelihood of the cascade effect being triggered is largely context-dependent. In contexts in which mental illness is stigmatized (leading to pathogenic vulnerabilities) and care is inadequate, or is dependent on the availability of resources, having a psychiatric disorder is more likely to involve cascading vulnerability. While there is rightly trepidation regarding the involvement of psychiatric inpatients in research, it is nevertheless important that the focus should be on identifying how a particular research context might trigger a cascade effect and how this may be addressed, rather than unreflexively evaluating the cascade potential and thereby ruling out recruitment among psychiatric inpatients.

In the context of genetic research involving psychiatric inpatients, the potential cascade vulnerability would be the fact that inpatients are wholly dependent on a particular institution for their care. They are therefore more at risk of being taken advantage of, in some way, due to the structural dimensions of institutionalization. Whether this state of dependency triggers a cascade of vulnerabilities will be strongly informed by the quality of ethics oversight and the strength of the research protocol to anticipate challenges that may arise, particularly regarding deferential vulnerability, as well as by the sensitivity of the researchers in the field. However, part of the cascade effect that must be considered is the way in which being in a situation of dependency leads to undue and disempowering forms of paternalism, even if well-intentioned. Indeed, one of the important advances in psychiatric care of psychotic disorders has been the recognition that patient agency and empowerment are a vital part of recovery.^65^

## 6 Concluding Remarks

In conclusion, we can infer clear duties expected of RECs and researchers in the case of considering and addressing vulnerability. RECs have a duty to assess the particularities of research contexts to ensure that protocols include adequate protections in order to minimize risks to participants, thereby permitting good research to take place. Such protections include ensuring that consent processes are appropriately tailored to specific contexts and potential impacts on decisions to freely participate have been considered. RECs have an equally important duty to ensure that potential participants are not needlessly excluded from studies as a form of special protection. Researchers are obligated to ensure that they are fully informed about various potential layers of vulnerability in particular research contexts and are reflexive in their responses to these challenges throughout the research process.

Our discussion shows that tensions around the ethics of psychiatric inpatient inclusion in research involve complex, intersecting factors and assumptions. Further, we have showed that a subset of these ethical concerns takes on particular weight in the context of genetic research in LMICs. Underlying beliefs about the moral status of institutionalized or hospitalized persons with psychiatric disorder promote ambivalence about inclusion of such persons in research. In this paper we have not drawn a normative conclusion about the rightness or wrongness of inclusion of psychiatric inpatients; rather, we have attempted to clarify the source of the ambivalence by identifying and analysing underlying assumptions of vulnerability in the context of the REC framework. In the case of inpatient participation in genetic research, we have suggested that layers of vulnerability associated with exploitation warrant particular attention, and that exploitation is a particular risk in LMICs. However, attempts to protect persons on the basis of a greater likelihood of exploitation should not entail automatic exclusion. As other scholars have argued, exploitation concerns can potentially be balanced by collection of viewpoints of parties deemed to be at risk of exploitation. Patient-centred medicine supports the view that patients should have opportunities to be involved in decision-making pertaining to their health and treatment in clinical contexts; and patients should also be given more opportunities to participate in the research that informs those decisions. There is ethical force to the argument about research participation,^66^ particularly in LMICs where ‘exploitation’ as a type of vulnerability could result in paternalistic exclusion of certain populations. Moreover, some of the reasons given by participants in support of participation in genetic research; e.g. being in a position to help others and the sense of empowerment that this may afford are particularly relevant for psychiatric inpatients,^67^ although more studies of this kind are needed in LMICs. Exclusion of psychiatric inpatients as research participants should therefore not be regarded as self-evident.

## Footnotes

1 Participation in genetic studies involves the provision of a sample from a participant that contains genetic material such as blood, saliva or hair samples. While participation in such studies poses minimal risk of direct, immediate harm to participants, there may be indirect risk. For example, a study found that genetic research that generates data about particular ethnic groups or subpopulations may exacerbate ethnic group stigmatisation, however findings indicate that this risk only applies in cases where groups or populations are already stigmatized (de Vries, J., Jallow, M., Williams, T.N. et al. (2012). Investigating the potential for ethnic group harm in collaborative genomics research in Africa: Is ethnic stigmatisation likely? *Social Science and Medicine.* 75(8), 1400-1407). The main concern is that genetic data which indicates that certain ethnic groups have genetic susceptibility or resistance to particular diseases, disorders or traits, may be misinterpreted with the result that perceptions of differences between groups are entrenched.

2 Venigalla, H., Mekala, H.M., Hassan, M., et al. (2017). An Update on Biomarkers in Psychiatric Disorders – Are we aware, Do we use in our clinical practice? *Mental Health in Family Medicine.* 13, 471-479. McGorry, P. Keshavan, M., Goldstone, S. et al. 2014. Biomarkers and clinical staging in psychiatry. *World Psychiatry.* 13(3), 211-223.

3 Weiss Roberts, L., Warner, T., & Brody, J. (2000). Perspectives of Patients With Schizophrenia and Psychiatrists Regarding Ethically Important Aspects of Research Participation. *American Journal of Psychiatry.* 157, 67. Schafer, I., Burns, T., Fleischhacker, W.W., et al. (2011). Attitudes of patients with schizophrenia and depression to psychiatric research: a study in seven European countries. *Soc Psychiatry Psychiatr Epidemiol.* 46, 159-65. Magyar, M.S., Edens, J.F., Epstein, M., et al. (2012). Examining attitudes about and influences on research participation among forensic psychiatric inpatients. Behav Sci Law. 30, 69-86. As these studies took place in high income countries, there is therefore a need for more studies in low- and middle-income countries.

4 Canadian Institutes of Health, National Sciences and Engineering Research Council of Canada & Social Sciences and Humanities Research Council of Canada. (2014). Tri-Council Policy Statement: ethical Conduct for Research Involving Humans. Retrieved March 19, 2019, from http://www.pre.ethics.gc.ca/eng/policy-politique/initiatives/tcps2-eptc2/chapter1-chapitre1/ch1_en. Council for International Organisations of Medical Sciences (CIOMS) & the World Health Organization (WHO). (2016). International Ethical Guidelines for Health-related Research Involving Humans. Geneva CIOMS.

5 Use of the term IRB is mostly limited to the United States, while the term REC is more commonly used internationally. We therefore use the latter term throughout our paper.

6 NHS Confederation. (2012). Defining mental health services: Promoting effective commissioning and supporting QIPP. N. Confederation, ed. United Kingdom.

7 Goffman. E. (1990). *Asylums : essays on the social situation of mental patients and other inmates.* New York: Doubleday.

8 Chow, W.S., & Priebe, S. (2013). Understanding psychiatric institutionalization: a conceptual review. *BMC Psychiatry.* 13, 169-169.

9 Goffman, op. cit. note 7.

10 Chow & Priebe, op.cit. note 8.

11 Priebe, S., Hoffmann, K.., Isermann, M., et al. (2002). Do long-term hospitalised patients benefit from discharge into the community? *Social Psychiatry and Psychiatric Epidemiology.* 37, 387-392; Segal, S., & Moyles, E. (1979). Management style and institutional dependency in sheltered care. *Social psychiatry.* 14, 159-165.

12 Chow & Priebe, op.cit. note 8.

13 Foucault, M. (2001). *Madness and civilization : a history of insanity in the Age of Reason.* London: Routledge.

14 Sisti, D., Segal, A., & Emanuel, E. (2015). Improving Long-term Psychiatric Care: Bring Back the Asylum. *JAMA.* 313, 243.

15 Ibid.

16 The National Commission for the Protection of Human Subjects of Biomedical and Behavioral Research. (1979). The Belmont Report: Ethical Principles and Guidelines for Biomedical Research Involving Human Subjects Washington DC.

17 Bracken-Roche, D., Bell, E., Macdonald, M.E., & Racine, E. (2017). The concept of ‘vulnerability’ in research ethics: an in-depth analysis of policies and guidelines. Health Res Policy Syst, 15(1), 8. W.M. Association. (2001). *World Medical Association Declaration of Helsinki: ethical prinicples for medical research involving human subjects* Retrieved March 19, 2019, from https://www.who.int/bulletin/archives/79%284%29373.pdf.

18 Carpenter, J.W.T., Gold, J.M., Lahti, A.C., et al. (2000). Decisional Capacity for Informed Consent in Schizophrenia Research. *Archives of General Psychiatry.* 57, 533; Kovnick, J.A., Appelbaum, P.S., Hoge, S.K., et al. (2003). Competence to consent to research among long-stay inpatients with chronic schizophrenia. *Psychiatric services.* 54, 1247-1252; Moser, D.J., Reese, R.L., Hey, C.T., et al. (2006). Using a brief intervention to improve decisional capacity in schizophrenia research. *Schizophr Bull.* 32, 116-20.

19 Luna. F. (2009). Elucidating the Concept of Vulnerability: Layers Not Labels. *International Journal of Feminist Approaches to Bioethics.* 2, 121-139. Kipnis, K. (2001). Vulnerability in research subjects: A bioethical taxonomy. In *Ethical and policy issues in research involving human participants*. Bethesda, MD: National Bioethics Advisory Commission; Mackenzie, C., Rogers, W., & Dodds, S (Eds.). (2014). *Vulnerability: New Essays in Ethics and Feminist Philosophy.* New York Oxford University Press.

20 CIOMS, op. cit. note 4.

21 McDonald, K.E. (2012). “We want respect”: adults with intellectual and developmental disabilities address respect in research. *Am J Intellect Dev Disabil.* 117, 263-74; Charlton, J.I. (1998). *Nothing about us without us : disability oppression and empowerment.* Berkeley: University of California Press. Carey, E., Griffiths, C. (2017). Recruitment and consent of adults with intellectual disabilities in a classic grounded theory research study: ethical and methodological considerations. *Disability & Society.* 32, 193-212.

22 Levine, C., Faden, R., Grady, C., et al. (2004). Consortium to Examine Clinical Research. The limitations of “vulnerability” as a protection for human research participants. *Am J Bioeth.* 4, 44-9.

23 Luna. F. (2018). Identifying and evaluating layers of vulnerability - a way forward. *Dev World Bioeth* 2018; Hurst, S.A. (2008). Vulnerability in Research and Health Care; Describing the Elephant in the Room? *Bioethics.* 22, 191-202.

24 DeMarco, J.P. (2004). Vulnerability: A Needed Moral Safeguard. *The American Journal of Bioethics.* 4, 82-84.

25 CIOMS, op. cit. note 4.

26 Schroeder, D., & Gefenas, E. (2009). Vulnerability: Too Vague and Too Broad? *Cambridge Quarterly of Healthcare Ethics.* 18(2), 113-121; Hoffmaster, B. (2006). What Does Vulnerability Mean? *Hastings Center Report.* 36(2), 38-45; Wendler, D. (2017). A pragmatic analysis of vulnerability in clinical research. *Bioethics.* 31(7), 515-525.

27 Luna, F. (2015). Rubens, Corsets and Taxonomies: A Response to Meek Lange, Rogers and Dodds. *Bioethics.* 29, 448-450.

28 Meek Lange, M., Rogers, W., & Dodds, S. (2013). Vulnerability in Research Ethics: a Way Forward. *Bioethics.* 27, 333-340.

29 Luna, op. cit. note 19.

30 Luna, op. cit. note 23.

31 Luna, op. cit. note 23.

32 Luna, op. cit. note 23.

33 Meek Lange et al., op. cite. Note 28.

34 Luna, op. cit. note 23; Rogers, W., Mackenzie, C., & Dodds, S. (2012). Why bioethics needs a concept of vulnerability. International Journal of Feminist Approaches to Bioethics. 5(2), 11-38.

35 Bracken-Roche, D., Bell, E., & Racine, E. (2016). The “Vulnerability” of Psychiatric Research Participants: Why This Research Ethics Concept Needs to Be Revisited. *Can J Psychiatry.* 61, 335-9.

36 Appelbaum, P.S. (2006). Decisional capacity of patients with schizophrenia to consent to research: taking stock. *Schizophr Bull.* 32, 22-5.

37 Ibid. Spencer, B.W.J., Gergel, T., Hotopf, M., et al. (2018). Unwell in hospital but not incapable: cross-sectional study on the dissociation of decision-making capacity for treatment and research in in-patients with schizophrenia and related psychoses. *Br J Psychiatry.* 213, 484-489.

38 Mintz, J., Sergi, M., Wirshing, D. (2005). A videotape intervention to enhance the informed consent process for medical and psychiatric treatment research. *American Journal of Psychiatry.162.* 186-188.

39 Carpenter et al., op. cit. note 18. Moser et al., op. cit. note 18. Jeste, D.V., Palmer, B.W., Golshan, S., et al. (2009). Multimedia consent for research in people with schizophrenia and normal subjects: a randomized controlled trial. *Schizophrenia bulletin.* 35, 719.

40 Campbell, M.M., Susser, E., Mall, S., et al. (2017). Using iterative learning to improve understanding during the informed consent process in a South African psychiatric genomics study. *PLoS One.* 12: e0188466. Further studies are required as there have been limited studies of this nature in African contexts.

41 Gergel, T. & Owen, G.S. (2015). Fluctuating capacity and advance decision-making in Bipolar Affective Disorder - Self-binding directives and self-determination. *Int J Law Psychiatry.* 40, 92-101.

42 We use the phrase ‘vulnerability bias’ to refer to whole-group exclusion from a protocol on the basis of an assumption of uniform vulnerability (in this case the assumption that there is a lack of capacity to consent) that is unwarranted and results in overprotection.

43 Horn, L., Sleem, H., & Ndebele, P. (2014). Research Vulnerability. In P. Ndebele and M. Horn (Eds.), *Research Ethics in Africa* M. Kruger, Stellenbosch: African SUN MeDIA; Emanuel, E.J., Wendler, D., Killen, J., et al. (2004). What makes clinical research in developing countries ethical? The benchmarks of ethical research.(Perspective). *Journal of Infectious Diseases.* 189, 930.

44 Ferguson, B. (2015). The Paradox of Exploitation. *Erkenntnis.* 81, 951-972; Emanuel, E. (2007). The paradox of exploitation: The poor exploiting the rich. In J. Lavery, C. Grady, E. Wahl and E. Emanuel, (Eds.), *Ethical issues in international biomedical research: A casebook.* New York Oxford University Press; Macklin, R. (2003). Bioethics, Vulnerability, and Protection. *Bioethics.* 17, 472-486.

45 In such cases, however, while there may be no obvious harm involved, and the situation may appear to be beneficial for the persons in question, the structures that sustain such exploitative conditions are inherently unjust. This insight is well captured by Sen who argues that our desires and preferences, and thus our sense of well-being, are largely determined by what we take to be possible given our “situation and station...[and are thus] compromises with reality” (Sen. A. (1985). Well-Being, Agency and Freedom: The Dewey Lectures 1984. Journal of Philosophy. 82(4), 169-221).

46 Kipnis, op. cit. note 19.

47 Kipnis, op. cit. note 19.

48 Zwolinski, M. & Wertheimer, A. (2017). *Exploitation.* Retrieved March 19, 2019, from https://plato.stanford.edu/archives/sum2017/entries/exploitation/.

49 Emanuel et al., op. cit. note 44. Macklin, op. cit. note 44. Hawkins, J.S., & Emanuel, E.J. (Eds.). (2008). *Exploitation and developing countries : the ethics of clinical research*. Princeton, N.J: Princeton University Press.

50 Walsh, A., Brugha, R., & Byrne, E. (2016). “The way the country has been carved up by researchers”: ethics and power in north-south public health research. *Int J Equity Health.* 15, 204.

51 Klitzman, R.L. (2012). US IRBS Confronting Research in the Developing World. *Developing World Bioethics.* 12, 63-73.

52 Ibid.

53 Gikonyo, C, Bejon, P, Marsh, V., et al. (2008). Taking social relationships seriously: lessons learned from the informed consent practices of a vaccine trial on the Kenyan Coast. *Soc Sci Med.* 67, 708-20; Tindana, P.O., Kass, N., Akweongo, P. (2006). The Informed Consent Process in a Rural African Setting: A Case Study of the Kassena-Nankana District of Northern Ghana. *IRB: Ethics & Human Research.* 28, 1-6; Tekola, F., Bull, S.J., Farsides, B., et al (2009). Tailoring consent to context: designing an appropriate consent process for a biomedical study in a low income setting. *PLoS Negl Trop Dis.* 3, e482.

54 Mbiti, J.S. (1990). *African religions & philosophy.* Oxford: Heinemann; Gyekye, K. (2011). *African Ethics*. Retrieved March 19, 2019, from https://plato.stanford.edu/entries/african-ethics/; Menkiti, I.A. (1979). Person and Community in African Traditional Thought. In *African philosophy : An introduction*. R.A. Wright, ed. Washington, D.C: University Press of America; Musana, P. (2018). The African Concept of Personhood and its Relevance to Respect for Human Life and Dignity in Africa and the Global Context. *African study monographs Supplementary issue.* 56, 21-32.

55 Dhai, A. (2018). The Life Esidimeni tragedy: Moral pathology and an ethical crisis. *S Afr Med J.* 108, 382-385.

56 Kipnis, op. cit. note 19.

57 Horn et al., op. cit. note 43.

58 Egbe, C.O., Brooke-Sumner, C., Kathree, T., et al. (2014). Psychiatric stigma and discrimination in South Africa: perspectives from key stakeholders. *BMC Psychiatry.* 14, 191; Kapungwe, A., Cooper, S., Mwanza, J., et al. (2010). Mental illness - stigma and discrimination in Zambia. University of Cape Town; Gureje, O., Lasebikan, V.O., Ephraim-Oluwanuga, O., et al. (2005). Community study of knowledge of and attitude to mental illness in Nigeria. *The British journal of psychiatry : the journal of mental science.* 186, 436; Shibre, T., Negash, A., Kullgren, G., et al. (2001). Perception of stigma among family members of individuals with schizophrenia and major affective disorders in rural Ethiopia. *Social Psychiatry and Psychiatric Epidemiology.* 36, 299-303; Shah, A., Wheeler, L., Sessions, K., et al. (2017). Community perceptions of mental illness in rural Uganda: An analysis of existing challenges facing the Bwindi Mental Health Programme. *Afr J Prim Health Care Fam Med.* 9, e1-e9; Opare-Henaku, A. & Utsey, S.O. (2017). Culturally prescribed beliefs about mental illness among the Akan of Ghana. *Transcult Psychiatry.* 54, 502-522.

59 Parrott, R., Kahl, M.L., Ndiaye K., et al. (2012). Health communication, genetic determinism, and perceived control: the roles of beliefs about susceptibility and severity versus disease essentialism. *J Health Commun.* 17, 762-78; Gould, W.A. & Heine, S.J. (2012). Implicit essentialism: genetic concepts are implicitly associated with fate concepts. *PLoS One.* 7, 38176; Dar-Nimrod, I. & Heine, SJ. (2011). Genetic essentialism: on the deceptive determinism of DNA. *Psychol Bull.* 137, 800-18.

60 Angermeyer, M.C., Holzinger, A., Carta, M.G., et al. (2011). Biogenetic explanations and public acceptance of mental illness: systematic review of population studies. *Br J Psychiatry.* 199, 367-72.

61 Humphreys, K., Blodgett, J.C., & Weiss Roberts, L. (2015). The exclusion of people with psychiatric disorders from medical research. *Journal of Psychiatric Research.* 70, 28-32.

62 It is interesting to note that it has been argued by Nickel that for this very reason, persons with serious illness are particularly vulnerable in research contexts (Nickel, P. (2006). Vulnerable Populations in Research: The Case of the Seriously Ill. *Theoretical Medicine and Bioethics.* 27(3), 245-264). In other words, the options of such persons to freely participate in research may be limited because of unwanted illness. In cases where research is believed to hold the promise of ultimately curing or alleviating their illness, such persons may be more susceptible to pressures to participate in research.

63 Weiss Roberts et al., op. cit. note 3.

64 However, assessing the potential benefits of genetic research is a complex endeavour. While exploratory genetic research may not directly lead to new treatments, research findings may support further pharmacogenetics research on, for example, the implications of particular genetic variants on drug metabolism. Such research is likely to lead to better clinical guidelines regarding, for example, how to treat fast and slow metabolisers.

65 Leamy, M., Bird, V., Le Boutillier, C., et al. (2011). Conceptual framework for personal recovery in mental health: systematic review and narrative synthesis. Br J Psychiatry. 199, 445-52.

66 Tinetti, M.E. & Basch, E. (2013). Patients’ responsibility to participate in decision making and research. *JAMA.* 309, 2331-2.

67 Cohn, E.G., Husamudeen, M., Larson, E.L., et al. (2015). Increasing participation in genomic research and biobanking through community-based capacity building. *J Genet Couns.* 24, 491-502; Sanderson, S.C., Linderman, M.D., Suckiel, S.A., et al. (2015). Motivations, concerns and preferences of personal genome sequencing research participants: Baseline findings from the HealthSeq project. *European Journal Of Human Genetics.24,* 14; Bui, E.T., Anderson, N.K., Kassem, L., et al. (2014). Do participants in genome sequencing studies of psychiatric disorders wish to be informed of their results? A survey study. *PLoS One.* 9, e101111.

